# Variability of the trophic state in a coastal reef system associated with submarine groundwater discharge in the Mexican Caribbean

**DOI:** 10.1007/s11356-024-32818-9

**Published:** 2024-03-20

**Authors:** Karla Camacho-Cruz, María Concepción Ortiz-Hernández, Laura Carrillo, Alberto Sánchez

**Affiliations:** 1 Instituto Politécnico Nacional, Centro Interdisciplinario de Ciencias Marinas, Avenida IPN, s/n Colonia Playa Palo de Santa Rita, C.P. 23096, La Paz, Baja California Sur Mexico; 2 Instituto de Ciencias del Mar y Limnología, Universidad Nacional Autónoma de México, Prolongación Av. Niños Héroes s/n, C.P. 77580 Puerto Morelos, Quintana Roo, Mexico; 3 El Colegio de la Frontera Sur, Unidad Chetumal Avenida Centenario Km 5.5, s/n Col. Pacto Obrero Campesino Chetumal, C.P. 77014, Quintana Roo, Mexico

**Keywords:** Karydis Index, δ^15^N, Anthropogenic pollution, Karst, Nitrates

## Abstract

Submarine groundwater discharges (SGD) have been associated with important sources of nutrients between the land and oceans that can generate eutrophication conditions. This study aims to analyze the behavior of nitrogen and phosphorus using the mixing curve method, to examine the variation of the trophic state using the Karydis Index, and to evaluate the δ^15^N in benthic organisms to trace the origin of nitrogen in neap tide (November) and spring tide (January) in the Manatí Cenote, and Nohoch-Teek reef lagoon in the Mexican Caribbean. Nitrogen and phosphate enrichment was in the Manatí Cenote during neap and spring tides. This enrichment was particularly noticeable in the reef lagoon during low tides in the areas influenced by SGD. In the Cenote, differences in the nitrate trophic state were observed, indicating an eu-mesotrophic condition during neap tide and a mesotrophic condition during spring tide. However, no significant differences were observed for ammonium (oligo-mesotrophic), nitrites, or phosphate compounds (oligotrophic). The trophic state reef lagoon exhibited a similar pattern but with different spatial variations. In both systems, phosphorus was a limiting nutrient, while δ^15^N suggested anthropogenic nitrogen uptake by several benthic organisms.

**Supplementary Information:**

The online version contains supplementary material available at 10.1007/s11356-024-32818-9.

## Introduction

Coastal coral reef systems provide some of the most valuable ecosystemic services. Their biodiversity supports fisheries and a large tourism industry (Nagelkerken et al. 2002). However, these systems worldwide are considered among the most sensitive, under pressure, and vulnerable (Ban et al. 2014), mainly due to anthropogenic pollution (Rey-Villiers et al. 2020). The Mesoamerican Reef System is the second largest barrier reef in the world, located on the Caribbean Mexican coast. Between 2015 and 2022, population growth and tourism increased by 24% and 139%, respectively (Secretaría de Turismo 2022; INEGI 2022). This resulted in a wastewater increase, of which only 59% was treated (CONAGUA 2022).

Submarine groundwater discharge (SGD) has become an essential source of chemical compounds, e.g., nutrients, pollutants, and metals, to coastal marine ecosystems that could alter the ecological status of water bodies (Moore and Arnold 1996; Slomp and Van Cappellen 2004; Paytan et al. 2006; Young et al. 2008; Tait et al. 2014; Oehler et al. 2019; Santos et al. 2021). In Quintana Roo, the lack of wastewater treatment poses a threat to coastal waters. This is particularly concerning due to the rocky bed composed of karstic limestone, characterized by triple porosity, and rapid and efficient infiltration that recharges the coastal aquifer (Beddows 2004; Bauer-Gottwein et al. 2014). When the coastal aquifer is positively hydraulically connected to the sea, groundwater can flow directly to the coast through SGD (Taniguchi et al. 2002; Burnett et al. 2003). Groundwater moves rapidly toward the coast, transporting inorganic nutrients and other substances from untreated sewage and failing septic systems. In the Mexican Caribbean, the average SGD has been estimated at 112 x 10^6^ m^3^ km^−1^ yr^−1^ (Null et al. 2014). Furthermore, a substantial freshwater discharge into the ocean along the coast has been reported, estimated to be as high as 650 cubic meters per second (Carrillo et al. 2016). Generally, the carried water is enriched in N, with an N:P ratio higher than the Redfield ratio, given that in carbonate systems, P immobilization through sediments is more efficient than N (Redfield 1934; Slomp and Van Cappellen 2004; Hernández-Terrones et al. 2011; Null et al. 2014). Therefore, P is considered limited in the Mexican Caribbean and other karstic environments, such as the Mediterranean Sea (Krom et al. 1991). Conversely, N is more stable in the nitrate form due to carbonates in the sediment and high oxygen availability, which reduces denitrification conditions (Rodellas et al. 2018). This makes this nutrient predominant in all coastal sites with SGD (Slomp and Van Cappellen 2004; Paytan et al. 2006; Wang et al. 2018).

Groundwater input and nutrient discharge vary significantly from site to site, and each area varies locally from one tidal cycle to another (Paytan et al. 2006; Wang et al. 2018). The significant quantities of anthropogenic or natural nitrogen influx into coastal waters can cause eutrophication processes and algae growth which limit light and oxygen availability (Lapointe et al. 2005; Paytan et al. 2006); it has also been shown to be an ecological modification factor of coastal ecosystems (Valiela et al. 1992). This nutrient influx from SGD into coastal waters can transport the nutrients kilometers away from their sources (Carruthers et al. 2005), which modifies the nutrient concentration and chemical speciation along the SGD paths (Santos et al. 2009; Montiel et al. 2018; Chen et al. 2020). However, its distribution will not only be determined by the physical processes but also be affected by different biological and physical-chemical processes (Broche et al. 1998). The nutrient influx from land into the Mexican Caribbean Sea could lead to serious environmental problems, as reef lagoons thrive in low-nutrient concentrations, where even slight increases may cause significant changes (Lapointe and Clark 1992). In the Mexican Caribbean Sea, there is a baseline of sensitive nitrate load indicators, δ^15^N in seagrass and octocorals in some coastal sites that can be used to trace the origin of nitrogen (natural deposition 0–1‰ or anthropogenic > 6‰) (Carruthers et al. 2005; Mutchler et al. 2007, 2010; Sánchez et al. 2013; Camacho-Cruz et al. 2020; Sánchez et al. 2023).

There are worldwide studies to evaluate the SGD nutrient input to the coast such as in Indonesia, the Mediterranean Sea, and the Southern China Sea (Garcia-Solsona et al. 2010; Liu et al. 2017; Montiel et al. 2018; Oehler et al. 2018, 2019; Wang et al. 2018; Bejannin et al. 2020). However, in the Mexican Caribbean, trophic state variations in reef lagoons influenced by SGD have not been completely studied due to (1) the complex interaction of physicochemical and biological ecosystemic processes and (2) the complex location of the groundwater mass connected to the coastal SGD and its monitoring. Favorably, in the Riviera Maya, Mexican Caribbean, some open cenotes (groundwater bodies) meander through the coastal mangrove swamp a few meters to the shoreline with an SGD connection with the coast. In this kind of system, the water exchange and its compounds between the subterranean aquifer and the sea are directly modulated mainly by sea level variations (Beddows 2004; Wang et al. 2018; Selvam et al. 2022). The following questions are posed: (1) What processes determine N and P distribution from spring to neap tide in a reef lagoon and a groundwater body (Cenote) of the Mexican Caribbean? (2) How does the behavior of the N:P ratio vary from spring to neap tide? (3) How does the trophic state vary from spring to neap tide? (4) Is anthropogenic nitrogen being assimilated by benthic organisms? To answer these questions, this study proposes to analyze the behavior of N and P through the mixing curve method and to examine the trophic state variation using the Karydis Index and also to evaluate the δ^15^N in benthic organisms to trace the origin of nitrogen at neap (November) and spring tide (January) in the Manatí Cenote and Nohoch-Teek reef lagoon in the Mexican Caribbean.

## Materials and methods

### Study site

**Fig. 1:**
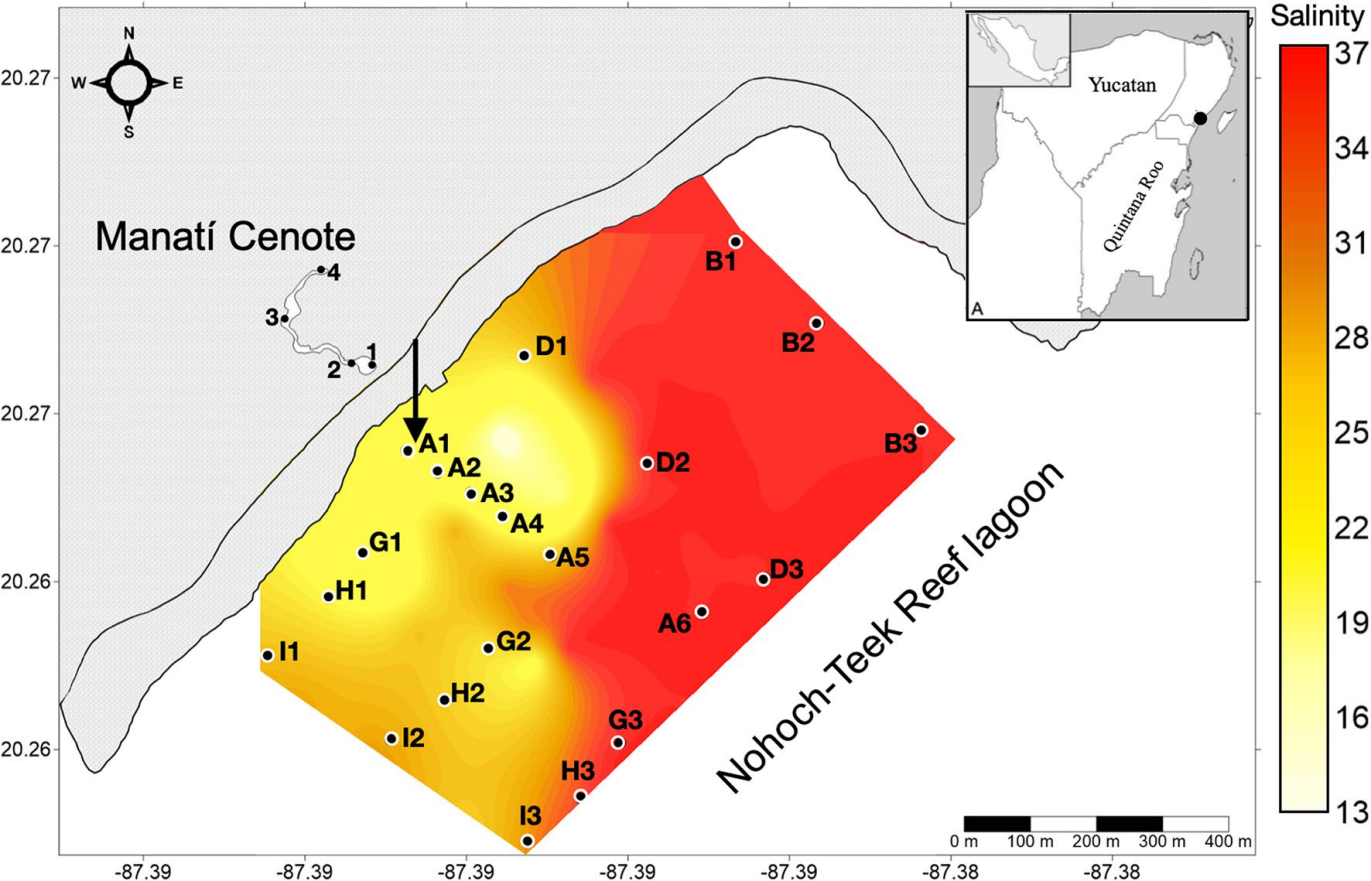
Study area in Quintana Roo, Mexican Caribbean. Nohoch-Teek reef lagoon and Manatí Cenote sampling stations and superficial salinity distribution for low tide of spring tide on 25 January 2020. The black arrow shows the SGD-Teek

The Nohoch-Teek reef lagoon (Fig. 1A) in the Mexican Caribbean is located in a sequence of Quaternary carbonate rocks (Beddows 2004). This reef lagoon receives groundwater from a shallow submarine spring, referred to as “SGD-Teek” in the present work. The lagoon has an ~3.5 m depth at ~46 m from the shoreline; the phreatic passage passes under the beach ridge with an extension of ~70 m, connected with the Manatí Cenote.

The Manatí Cenote receives an average of ~400 tourists per day (pers. comm. Manatí Cenote manager Alex). It is an open channel that meanders through the coastal mangrove swamp with an extension of ~250 m at the head. At the end of the open channel, it is connected by a network of phreatic passages that link through to the interior sections of the Nohoch Nah Chic System. These systems connect with one of the world’s biggest over-flooded cave systems, Sac Actun (Beddows 2004; Smart et al. 2006). Inland seawater flow into the open channel has been previously demonstrated during high tides at Manatí Cenote (Beddows 1999). Beddows (2004) states that sea level variation is the main control over the aquifer’s outflow, with annual freshwater outflow volume from the Manatí Cenote is −9.34 ± 2.87 × 10^7^ m^3^ yr^−1^; the velocity outflow and sea level are positively correlated.

Based on surface salinity maps (Fig. 1A), SGD-Teek is the only freshwater source entering the Nohoch-Teek reef lagoon. Thus, SGD-Teek is considered the largest pollutant source. However, there are few hotels and restaurants along the coastline, and the poor sewage system could also be a contamination source. However, it has been observed that some hotels and restaurants discharge their sewage into the coastal zone through punctual or diffusion points (pers. obs.).

The Mexican Caribbean has a tropical climate with two main seasons based on precipitation: a dry season (average rainfall of 124 mm) from March to June and a wet season (656 mm) from July to October. However, there is a period when precipitation decreases by almost half from November to February (average rainfall of 321 mm), characterized by the passes of cold fronts. This is called the “nortes” season (Parra et al. 2014). For most of the year, trade winds predominate north-east-southeast (3–9 m/s). During cold fronts, the wind (> 10 m/s) prevails in a north-northeast and northwest direction (Coronado et al. 2007; Carrillo et al. 2009). Tides in this region are mixed semidiurnal microtides, with spring tide amplitude of approximately 0.4 m, and neap tide amplitudes as small as 0.05 m; the maximum sea water levels are registered in September, October, and November (hurricane season), while minimum levels occur in early January and May–June.

### Physicochemical and biological sampling

The sampling arrangement at the Nohoch-Teek Reef Lagoon and Manatí Cenote is shown in Figure 1A, and specifications are shown in Table 1. Water samples were collected through November 23, 2019, during at neap tide, and through January 25, 2020, during a spring tide. Manatí Cenote station 1 (cenote mouth, beside coastal subterranean passage), a HOBO U20-001-01 Water Level and a HOBO U24-002 conductivity and temperature sensors were installed and programmed to record data every 15 minutes.

**Table 1:** Sampling sites, collection dates/times, and tidial periods for Reef lagoon and Cenote

Date/season	Data 1991–2021 average bimonthly precipitation* (mm)	Water	Hour sample	Tide	Period of tide	Transect/site
Nov 23 2019 / north	Oct–Nov 135.5	Coastal water (Lagoon reef)	9:22–11:25	Neap	High tide	A, B, D, G, H and I
			16:38–17:40		Low tide	A, B, D, and G
		Fresh water (Cenote)	8:22–12:30		High tide	1–4
			13:30–17:18		Low tide	1–4
Jan 25 2020 / north	Dec-Jan 58	Coastal water (Lagoon reef)	11:30–15:35	Spring	High tide	A, B, D, G and I
			16:00–17:27		Low tide	A, B, D, and G
		Fresh water (Cenote)	10:00–13:00		High tide	1–4
			14:00–17:36		Low tide	1–4

*Copernicus Climate Change Service, Tulum, Quintana Roo

Nutrient water samples for nitrite, nitrate, ammonium, phosphorous, and total phosphorous analysis were collected at the surface using 50 ml polypropylene bottles and preserved on ice. On July 15, 2019, and January 25, 2020, octocoral samples (2 cm, apical branch fragment) were extracted manually from an approximate depth of 3 m on the Mesoamerican reef bordering the Nohoch-Teek reef lagoon (Fig. 1A). Samples were deposited in plastic bags and preserved on ice, including *Gorgonia flabellum*, *Plexaura kükenthali*, *Eunicea flexuosa*, and *E. succinea*. On July 15, 2019, filamentous green algae were manually extracted from station 1 of the Manatí Cenote. The algae were carefully collected from rocky substrates, placed in plastic bags, and preserved on ice.

### Lab analyses

Water samples were transferred to the ECOSUR Chemistry Laboratory Unidad Chetumal in a cooler. 10 ml of unfiltered water was separated from each sample for total phosphorus analysis. Before analysis, the remaining samples were filtered with Whatman™ 0.45-μm microfiber filters and preserved at 4°C. Dissolved inorganic nutrients, NO_3_^−^ (nitrates), NO_2_^−^ (nitrites), NH_4_^+^ (ammonium), TP (total phosphorous), and PO_4_^3−^ (orthophosphates), were analyzed based on Strickland and Parsons (1968). The colorimetric readings were done with a visible-UV spectrophotometer SHIMADZU UV-1700. The detection limits were 0.16 μM l^−1^ for nitrates, 0.01 μM l^−1^ for nitrites, 0.15 μM l^−1^ for ammonium, and 0.05 μM l^−1^ for orthophosphates.

The octocoral and filamentous green samples were rinsed with deionized water and dried in an oven at 40°C for 48 h. Samples were macerated in an agate mortar, weighed 1 mg, and packed in tin capsules at the CICIMAR-IPN Chemistry Laboratory in La Paz, Baja California Sur. Samples were analyzed at the Center for Stable Isotopes at the University of New Mexico, US.

### Data analyses

The mixing curve method was used to analyze nitrogen and phosphorous behavior (Boyle et al. 1974), representing the chemical constituent concentration as a function of the conservative tracer distribution. These results are compared with the chemical constituent’s distribution in an ideal physical mixing. For this, nitrogen and phosphorous concentrations were represented as a function of chlorinity. As the method requires the definition of the ideal mixing line’s final members, stations 3 and 4 averages of the cenote obtained during the first morning hours were selected for the last freshwater members to avoid interference caused by the recreational use of the cenote (end members were chosen for each sample period, November and January). The average of stations B3, D3, A6, G3, H3, and I3 obtained during the first morning hours was used to select the end members corresponding to marine water, each corresponding to November and January. In the figures, the ideal mixing line is represented by a dashed line.

Salinity (‰) = 1.80655 × chlorinity (‰)

Nutrient concentrations were analyzed to estimate the trophic status according to the trophic index of Karydis et al. (1983). Eutrophication, a priority concern for various water bodies, has been quantified in recent decades due to nutrient inputs from submarine groundwater discharge. Standardizing assessment methods is challenging, and no single method fully represents eutrophication. Dimensionless indices, like the trophic index of Karydis et al. (1983), focus on specific nutrient conditions; it is sensitive to eutrophication stress, and simple data is obtained from the calculations:

$$\mathrm{TI}=\frac{C}{}C- logx+ logA$$

where TI is the trophic index per nutrient per sampling site during the study period, composed of *M* samples; *A* is the number of sampling stations during the study period; *C* is the log of the total nutrient load, that is the sum of *X*_*ij*_ concentrations of the nutrient obtained in each of the *A*_*i*_ stations during the *M*_*j*_ samplings; and *x* is the nutrient total concentration in a certain station.

In the Karydis Index scale, TI values more than 5 indicate a eutrophic state, values of TI between 5 and 3 indicate a mesotrophic state, and values of TI less than 3 indicate an oligotrophic state.

Data obtained by the Center for Stable Isotopes at the University of New Mexico, US, were used to investigate the nitrogen origin used by benthic organisms, where the isotopic nitrogen values were obtained and expressed in delta notation (‰) as indicated by the following equation:

$${\delta}^{15}\mathrm{N}\left({\permille} \mathrm{vs}.\mathrm{air}\right)=\left(\left(^{15}\mathrm{N}{/}^{14}{\mathrm{N}}_{\mathrm{sample}}{/}^{15}\mathrm{N}{/}^{14}{\mathrm{N}}_{\mathrm{std}}\right)\hbox{-} 1\right)\times 1000$$

These results are presented concerning of atmospheric N. The isotope analysis precision was <0.2‰.

Surfer 12 was used to plot spatial contours of the trophic status in the Nohoch-Teek reef lagoon with kriging interpolation due to its suitable linear unbiased prediction of the intermediate values in spatial analysis (Papritz and Moyeed 1999). Linear regressions were conducted between salinity and water depth, chlorinity and nitrate, chlorinity and ammonium, and chlorinity and total phosphorous.

## Results

**Fig. 2:**
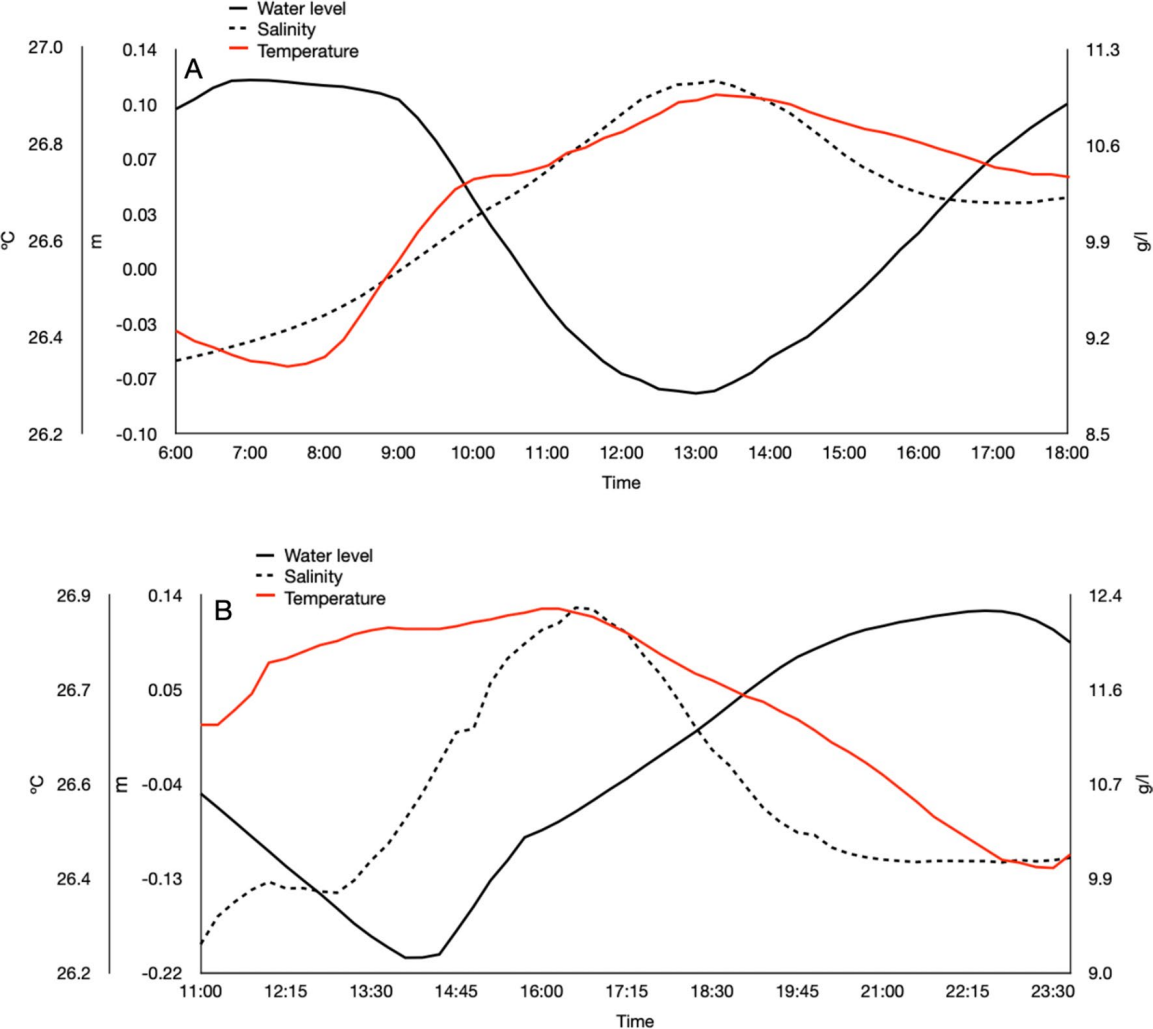
Water level, salinity, and temperature time-series at station 1 in the Manatí Cenote. **A** Neap tide on November 23, 2019. **B** Spring tide on January 25, 2020

Time series observations of salinity, water level, and temperature at station 1 in the Manatí Cenote are shown in Figure 2A, B. Neap tide was on November 23, 2019; the low tide was recorded at 13:45 h, with the highest salinity and temperature values (11.1 ± 0.05 g/l and 26.9 ± 0.01°C). The correlation between salinity and depth was negative (*R*= −0.92, *p*=0.0000). Spring tide was on January 25, 2020; the low tide was recorded at 15:00 h with the highest salinity and temperature values (12.2 ± 0.10 g/l and 27.16 ± 0.01°C). The correlation between salinity and depth was negative (*R*= −0.29, *p*=0.035).

### The behavior of dissolved inorganic constituents

**Fig. 3:**
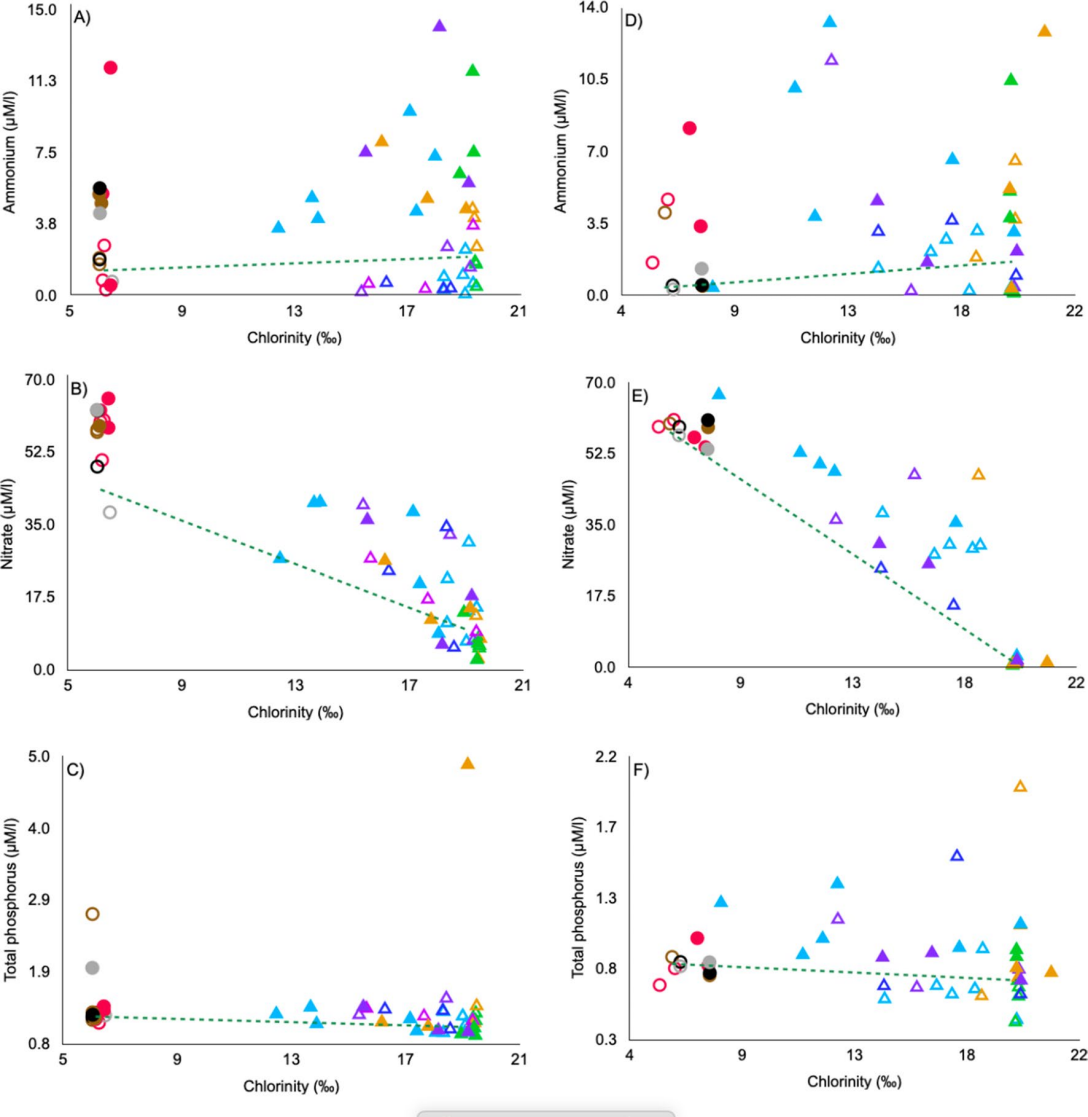
Mixing curve method, left side (**A–C**) represents neap tide data on November 23, 2019: empty and filled triangles represent reef lagoon data between 9:22–11:25 h and 16:38–17:40 h, respectively. Empty and filled circles represent Manatí Cenote data between 8:22–12:30 h and 13:30–16:51 h, respectively. The right side (**D–F**) represents spring tide data on January 25, 2020: empty and filled triangles represent reef lagoon data between 11:30–15:35 h and 16:00–17:27 h, respectively. Empty and filled circles represent Manatí Cenote data between 10:00–13:30 h and 13:30–17:37 h, respectively. To represent the sampled transects in the reef lagoon: light blue corresponds to transect A, green to transect B, orange to transect D, purple to transect G, pink to transect H, and dark blue to transect I. To represent the Manatí Cenote sites: red indicates station 1, brown indicates station 2, black indicates station 3, and gray indicates station 4

Figure 3 shows the relationship of chlorinity concerning ammonium, nitrate, and total phosphorus concentrations for the Nohoch-Teek reef lagoon and Manatí Cenote.

### Manatí Cenote

On November 23, during the initial morning sampling hours, ammonium concentrations at stations 1 and 4 were observed to fall below the Ideal Mixing Line (IML). A similar behavior was also observed at station 1 during the afternoon sampling session. In contrast, ammonium concentrations were above the IML during the rest of the day (Fig. 3A). Nitrate concentrations during the first hours of the day were below the IML at 4. Conversely, nitrate concentrations were above the IML during the rest of the study period (Fig. 3B). Total phosphorus concentrations were above the IML during the entire sampling period (Fig. 3C).

On January 25, 2020, ammonium concentrations corresponding to stations 3 and 4 in the time of the first morning sampling hours were below the IML, while stations 1 and 2 were positioned above the IML. In the course of the afternoon sampling, all the stations were located above the IML (Fig. 3D) except for station 3. Nitrate concentrations at station 1 were found to be below the Ideal Mixing Line (IML) during the morning; however, as the sampling progressed, all stations were consistently positioned above the IML (Fig. 3E). In the morning, total phosphorus concentrations at stations 3 and 2 exceeded the Ideal Mixing Line (IML). In the afternoon, stations 1 and 4 also exhibited concentrations above the IML (Fig. 3F).

### Nohoch-Teek reef lagoon

On November 23, ammonium concentrations were below the IML in 76 % of the sites sampled before midday. The remaining 24 % were positioned above the IML, corresponding to the stations near the barrier reef and transect D, adjacent to the SGD-Teek. In the afternoon, the ammonium concentration was above the IML in all stations (Fig. 3A). Nitrate concentrations decreased as chlorinity increased in 62% of the sites, corresponding to transects A, G, H, and I. Nitrate was above the IML before midday, but below the IML in the remaining 38% of the sites. In the afternoon, nitrate concentrations were located above the IML in 73 % of the sampled sites (transects A, D, and G, except for A6 and G3) (Fig. 3B). Total phosphorus concentrations were below the IML in all sampling sites (Fig. 3C).

On January 25, ammonium concentrations were above the IML in 55.5 % of the sites (transects A, D, G1, and I, except for A5, A6, and I3) during midday. In contrast, ammonium concentrations of all the sites were located above the IML in the afternoon (Fig. 3D), except stations A1 and D2. Nitrate concentrations in 72.2 % of the stations (transects A, D1,3, G, and I) monitored during midday were positioned above the IML, while all the stations were above the IML in the afternoon (Fig 3E). Total phosphorus concentrations at midday in 66.6 % of the sites (transects A, B, D1, G2, and I2, 3) were located below the IML. In the afternoon, all areas were above the IML (Fig. 3F).

### The trophic state of dissolved inorganic nutrients and N:P ratio

#### Manatí Cenote

On November 23, 2019, nitrate was eutrophic throughout the sampling period, except in station 2, where its behavior was mesotrophic in the morning. The ammonium condition went from oligotrophic in the morning to mesotrophic during the afternoon. Nitrites, total phosphorus, and orthophosphates showed an oligotrophic state throughout the sampling period. The N proportion was greater than that of P throughout the sampling (48.7 ± 10.9). On January 25, 2020, nitrate showed a mesotrophic condition throughout the sampling period. Nitrite concentrations were below the detection limit. Ammonium behaved mesotrophic, except in stations 1, 2, and 3 in the morning, where its condition was oligotrophic. Total phosphorus and orthophosphates showed an oligotrophic behavior throughout the sampling period. The N proportion exceeded P during the entire sampling (72.7 ± 14.8) (Table 2).

**Table 2:** N:P ratio across the sampling period in Reef lagoon and Cenote

Date/hour	23/Nov/2019 9:22–11:25	23/Nov/2019 16:38–17:40	25/Jan/2020 8:22–12:30	25/Jan/2020 11:30–17:18
Site	N:P (μM)			
A1	12.9	41.1	68.7	55.4
A2	17.7	24.6	54.4	72.4
A3	6.7	34.1	45.0	55.1
A4	13.6	40.8	36.4	46.0
A5	31.2	25.6	45.9	45.6
A6	14.8	16.8	2.6	5.4
B1	6.1	21.5	2.2	5.2
B2	5.1	15.1	1.5	16.6
B3	8.2	13.8	2.0	6.8
D1	7.5	30.9	83.1	7.3
D2	6.1	16.5	2.3	1.4
D3	16.3	4.0	7.4	18.4
G1	33.0	32.4	43.2	30.5
G2	24.3	24.3	73.1	40.9
G3	8.7	20.2	2.3	5.5
H1	21.1	ND	12.4	ND
H2	14.6	ND	41.3	ND
H3	11.4	ND	4.8	ND
I1	27.1	ND	ND	ND
I2	18.8	ND	ND	ND
I3	5.8	ND	ND	ND
Cenote 1	52.9	58.5	90.8	47.9
Cenote 2	51.7	63.2	81.3	60.1
Cenote 3	50.4	67.8	71.7	72.4
Cenote 4	38.5	66.0	81.5	66.4

#### Nohoch-Teek Reef Lagoon

**Fig. 4:**
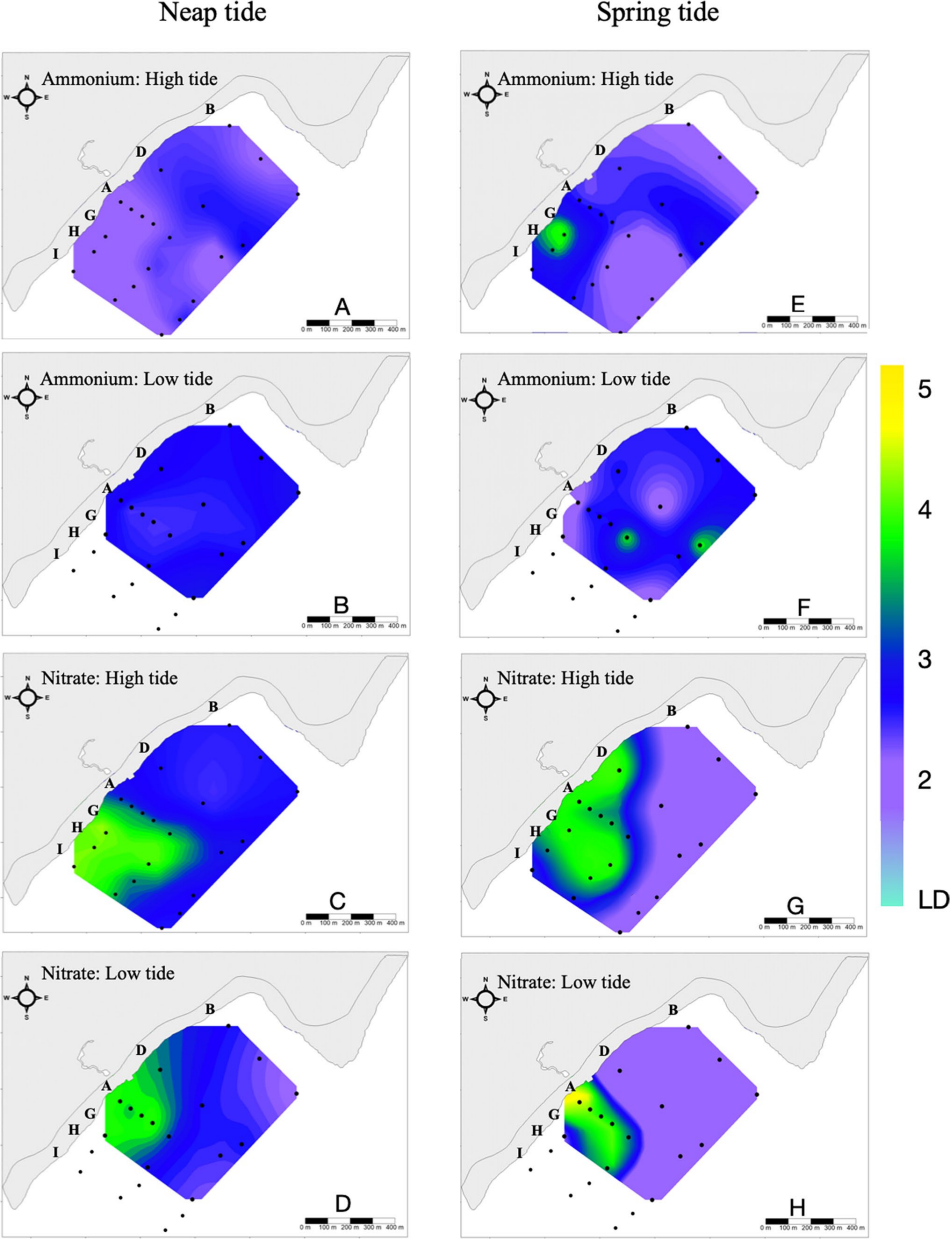
Distribution of Surface Eutrophication for ammonium and nitrate according to the Karydis et al. (1983) Index. Left side (**A–D**): neap tide data on November 23, 2019. Right side (**E–H**): spring tide data on January 25

On November 23, 2019, the N:P ratio (Table 2) showed a higher nitrogen proportion concerning P (23.7 ± 6.3) between 9:22 and 11:25 h in 38% of the stations, mainly those located southwest of the SGD-Teek. Nitrates (Fig. 4C) and total phosphorus (Fig. 1A, supplementary material) showed a mesotrophic condition based on the trophic state index. Nitrite condition showed an oligotrophic behavior (Fig. 2A, supplementary material), while ammonium was mesotrophic in 33% of the stations and oligotrophic in the rest of the reef lagoon (Fig. 4A). For orthophosphates, 38% of the stations presented mesotrophic conditions (A1, A3, A6, H, I1, and I3), while the rest of the reef lagoon was oligotrophic (Fig. 1C, supplementary material). Between 16:38 and 17:40 h, the N:P ratio showed a higher nitrogen proportion concerning phosphorus (27.4 ± 8.5) in 66% of the stations (transect A, D, and G) (Table 2). Based on the trophic state index, nitrates and ammonium showed a mesotrophic condition (Fig. 4B, D). Total phosphorus, orthophosphate, and nitrites showed an oligotrophic condition (Figs. 1B, D and 2B, supplementary material)

On January 25, 2020, the N:P ratio presented a higher N proportion concerning P (36.4 ± 16.4) between 11:30 h and 15:35 h in 50% of the stations (stations A1-5, D1, G1-2, and I2) (Table 2). Based on the trophic state index, nitrate was mesotrophic in 55.5% of all sites (stations A1-5, D1, G1-2, and I1-2). The rest of the reef lagoon presented an oligotrophic condition (Fig. 4G). The ammonium condition was mesotrophic in 44.4% of the stations (stations A2-4, D2-3, G1, and I1-2), while the rest of the reef lagoon showed an oligotrophic condition (Fig. 4E). Total phosphorus, orthophosphate, and nitrites showed an oligotrophic condition (Figs. 1E, G and 2C, supplementary material). The N:P ratio showed a higher N proportion concerning P (42.3 ± 18.1) between 16:00 and 17:27 h in 60% of the stations. Based on the trophic state index, nitrate showed a mesotrophic condition in 46.6% of all sites (A1-5, G1-2). In contrast, the rest of the reef lagoon showed oligotrophic conditions (Fig. 4H). Regarding ammonium, 73.3% of the sites (A2-6, B, D1-3, and G2) showed mesotrophic conditions, while the rest of the lagoon showed oligotrophic conditions (Fig. 4F). Total phosphorus, orthophosphate, and nitrites showed an oligotrophic condition (Figs. 1F, H and 2D, supplementary material).

### δ^15^N in benthic organisms

The filamentous algae collected from the cenote on July 15, 2019, had a δ^15^N value of 5.5‰. In comparison, values of the octocorals *G. flabellum* and *P. kükenthali* collected from the reef lagoon varied between 6.1 and 6.2‰, respectively. On January 25, 2020, δ^15^N values in *G. flabellum* varied between 5.9–6.2‰, while *E. succinea* presented a value of 6.5‰.

## Discussion

Nitrogen and P contribution from land to sea not only increases the concentrations of these elements in coastal waters but also alters the stoichiometric balance (Howarth 2008; Pavlidou et al. 2014; Santos et al. 2021), which can produce many ecological effects, including eutrophication acceleration (Wang et al. 2021). Globally, in a coastal karst zone, SGD have been recognized as essential nutrient sources for coastal systems (Taniguchi et al. 2002; Burnett et al. 2003; Paytan et al. 2006; Moore 2010). Understanding the origin of nutrients in a coastal karst zone influenced by submarine groundwater discharge is complex. This complexity arises from the intricate biogeochemical and transport processes that regulate nutrient dynamics, coupled with the diverse anthropogenic and natural sources. (1) In groundwater, N and P availability may be related to anthropogenic contributions (fertilizers, wastewater) or atmospheric deposition (Tiessen 1995; Nolan and Stoner 1995), where its variability will depend on the type of aquifer, aquifer permeability, recharge rate, and climate (Slomp and Van Cappellen 2004); (2) while the availability of N and P inputs to coastal surface water is influenced by physical and chemical processes occurring in the mixing/transition zone, such as flow and turnover rates, sea level variation, winds, bathymetry, density, redox conditions, primary production, and sediment sorption capacity (Slomp and Van Cappellen 2004; Kroeger and Charette 2008; Spiteri et al. 2008; Santos et al. 2021).

### Manatí Cenote

#### Behavior of N, P, and water exchange evidence

In general, the ammonium behavior shows clear enrichment during the afternoon of both neap and spring tide. This enrichment was particularly conspicuous at station 1, which was expected considering: (1) tourists spend the longest time engaged in aquatic activities around station 1 than in the rest of the cenote; (2) an underground cavern establishes communicates with the coastal region, potentially introducing seawater inputs containing organic matter; and (3) in stations 3 and 4, caverns connect with the underground aquifer, leading to distinctive mixing processes influenced by the redox conditions that arise. In contrast, the losses correspond to the neap tide’s high tide (8:00 h) at stations 2, 3, and 4. In the spring tide, the losses correspond to the data collected during midday and afternoon (low tide) at stations 3 and 4. Thus, chemical and biological factors like nitrification or assimilation processes by phytoplankton may be occurring in both cases. In addition, stations 3 and 4 are the furthest away from tourism and with the greatest influence of freshwater.

On November 23, during the neap tide, the dominant nitrate trend indicated enrichment, primarily associated with groundwater contributions enriched with nitrate and influenced by tourism. Typically, subterranean freshwater is nitrogen-enriched and exists in a more stable nitrate form due to the presence of carbonates in the sediment and high oxygen availability, which mitigates denitrification conditions (Rodellas et al. 2018), establishing it as the predominant nutrient. However, as the sea level began to decrease, some losses were recorded, potentially linked to a reduction in freshwater input influenced by physical tidal modulation or chemical and biological processes. In contrast, during the spring tide, nitrate behavior was generally conservative in almost all stations, registering some gains as the sea level began to increase. These gains are likely associated with the rising levels of subterranean freshwater. Total phosphorus showed a dominant trend registering enrichment, similar to nitrate, which may be due to tourist contribution, organic matter remineralization, or even due to the mixing between groundwater and seawater, which can generate desorption processes influenced by the speed and direction of water flow. (Romero et al. 2007; Wang et al. 2014). Particularly, during the spring tide, some losses were recorded, which could have been influenced by chemical or biological processes such as coprecipitation with dissolved Ca with sediments or phytoplankton absorption (Hernández-Terrones et al. 2011; Slomp and Van Cappellen 2004).

In the Manatí Cenote, the water flow direction (69.62 ± 1.55° dominated by outflow), the water volume discharged (−9.34 ± 2.87 × 10^7^ m^3^ yr^−1^), and the positive correlation of groundwater flow with sea level variation can explain the continuous nutrient transport to the coastal zone. The flow presents complex semidiurnal variation, having sea level variation as the principal control on aquifer outflow (Beddows 1999; Beddows 2004). Besides subterranean water influenced by anthropogenic contributions, also the precipitation could be a considerable factor in increasing the nutrient transport, or dilution, by the aquifer meteoric recharge. However Beddows (2004) found that the seasonal pattern of freshwater outflow is constant, with only 5% of reduction from the wet season to the dry season.

Throughout the observations conducted in this study, it was observed that the salinity and temperature of the Manatí Cenote exhibited a negative correlation with changes in sea level (*R*= −0.929, *p*= 0.000 neap tide; *R*= −0.292, *p*=0.035 spring tide). This corroborates what was previously observed by Beddows (2004) and supports a water exchange between the Cenote and the Reef lagoon, with a nutrient interchange under the control of semidiurnal tidal variations. Nevertheless, these findings underscore the importance of acquiring a comprehensive understanding of biogeochemical nutrient cycling in coastal karstic sediments under the influence of submarine groundwater discharge. This is crucial because physical forces alone do not constitute the primary controls on nutrients. Nutrient behavior can also be attributed to biogeochemical transformations taking place within the mixing zone, involving processes such as the interaction of fresh groundwater with seawater, biological uptake, sediment sequestration, or inputs driven by recirculation (Rodellas et al. 2018; Slomp and Van Cappellen 2004; Bejannin et al. 2020). The significant contribution of groundwater highlights the importance of the input of dissolved inorganic nutrients entering the coastal zone through SGD’s (Rodellas et al. 2018), with the potential to be exported to open sea.

#### Trophic status and N:P ratio

Total phosphorus, orthophosphates, and nitrites showed an oligotrophic behavior during both sampling periods. However, considering the phosphorus enrichment in the system registered in a major site (Fig. 3), oligotrophic future conditions could change to mesotrophic if tourism activities, and wastewater treatment are not regulated. Despite the low concentrations of phosphate in groundwater attribute to its rapid removal through sorption to ferroxides or coprecipitation with Al and Ca (Slomp and Van Cappellen 2004; El-Gamal et al. 2012; Null et al. 2012; Szymczycha et al. 2012; Pavlidou et al. 2014). The continuous input from the constant groundwater flow and tourist activities may exceed the sorption capacity of the system. With regard to nitrites, an oligotrophic state was expected, given the limited availability of nitrites resulting from their instability; they are rapidly oxidized to nitrates (Badee Nezhad et al. 2017). The eu-mesotrophic behavior displayed by nitrate and ammonium at most sites during both neap and spring tides aligns with the reported enrichment of these ions. Tourist activities in the cenote throughout the day may act as a direct source of ammonium, with peak concentrations observed in November and January (11.8 and 8.1 μM NH4, as reported by Camacho et al. in preparation). These concentrations are likely to undergo attenuation during transport to the aquifer, a process attributed to phenomena such as nitrification (DO 1–2 mg/l, Camacho-Cruz et al. 2020), mineralization, and sorption (Slomp and Van Cappellen 2004). Meanwhile, the continuous influx of groundwater could potentially serve as the primary source of nitrate, given its prevalence as the dominant form of nitrogen in groundwater, owing to its stability and incorporation through wastewater. Ávila-Torres et al. (2023) applied Carlson’s Trophic State Index to assess various trophic levels in cenotes subjected to different anthropogenic influences; however, no distinct patterns were identified. In cenotes located between the municipalities of Panaba and Dizilam in Yucatan, facing agricultural pressures, oligotrophic, eutrophic, and mesotrophic levels were observed. Conversely, cenotes in the municipality of Lazaro Cardenas, Quintana Roo, subjected to recreational activities pressure, exhibited eutrophic and mesotrophic levels. Apart from the diverse anthropogenic influences, cenote hydrodynamics significantly influence trophic conditions. Lentic cenotes tend to easily elevate trophic states, promoting organic matter production. In contrast, lotic cenotes are expected to have lower nutrient levels due to faster water turnover. The continuous flow in lotic cenotes also aids in vertically distributing nutrients, bringing nutrient-rich bottom waters to the surface (Schmitter-Soto et al. 2002). In the case of Manatí Cenote, the continuous outflow (69.62 ± 1.55°) could be a favorable factor in the trophic state.

If the outflow is disrupted, such as through cave collapse or obstruction by garbage, as is currently happening with the cave that connects to the coastal area of Manatí Cenote, the continuous influx of nitrogen may compromise the system. The gradual accumulation of nutrients could lead to eutrophication, disrupting the stoichiometric balance (Lapointe and Clark 1992; Tett et al. 2007; de Jonge and Elliott 2001; Wang et al. 2020). This trend was evident in our study, where the N:P ratio exceeded the Redfield ratio (48.7 ± 10.9).

#### Evidence of anthropogenic nitrogen

The nutrient contribution from anthropogenic influence has been documented in the Manatí Cenote for over a decade (Mutchler et al. 2010; Camacho-Cruz et al. 2020). Mutchler et al. (2010) reported to the Manatí Cenote, δ^15^N values of 5.5‰ in filamentous algae (*Derbesia* and *Polysiphonia*), suggesting a nitrogen source derived from untreated wastewater (Aravena et al. 1993). In the present study, the isotopic values in filamentous algae were similar to those reported by Mutchler et al. (2010). This suggests the presence of a continuous nitrogen anthropic source associated with tourist use in the cenote, or wastewater influx through subterranean water. Considering that the δ^15^N of untreated wastewater ranges from 5 to 9‰ (Aravena et al. 1993; Waldron et al. 2001).

According to stable isotope analyses of Mutchler et al. (2010), anthropogenic N sources contributed to nutrient loads in several cenote systems: *Cladophora* sp. have 6.2 ± 0.9‰ δ15N value in Beach cenote, 6.1‰ δ15N value in Bat Cave and Boodleopsis pusilla show 7.8‰ δ15N value in Pueblo Cave. Similar values have been recorded in filamentous algae from Yal Kú Lagoon (6 ± 0.3‰), suggesting the presence of terrestrial N inputs into the groundwater from septic systems, or untreated wastewater (Mutchler et al. 2007).

Nitrogen transformation by denitrification leads to ^15^N enrichment, and an increase in δ^15^N values of biological tracers (Mariotti et al. 1988), however, the length of residence times for groundwater may be important determinants for the relative importance of denitrification and isotopic signatures of nitrogen (Cole et al. 2006). Sites with longer residence times would be expected to have higher δ15N values (Mutchler et al. 2010). Given the predominant outflow of groundwater observed in Manatí Cenote (69.62 ± 1.55°, dominated by outflow) and the consistent δ15N values in filamentous algae since 2007, the nitrogen source is likely anthropogenic. Yet, lacking precise estimates of nitrogen transformations, evaluating the relative impact of denitrification and anthropogenic nitrogen sources on algal tissue δ15N values proves challenging. However, both processes probably contribute to the observed pattern.

### Nohoch-Teek reef lagoon

#### Behavior of dissolved inorganic constituents

Most sites, mainly in transects A, G, H, and I, were located above the ideal nitrate mixing line during neap tide (Fig. 3). Concentrations of NO_3_^−^ showed an inverse, linear correlation with salinity (*R*= ´0.74, *p*= 0.000), clearly suggesting (1) that enrichment principally could be influenced by the groundwater plume from SGD-Teek and (2) that conservative mixing in the coastal aquifer. In Figure 1, the values of surface salinity can be observed, which demonstrate the presence of groundwater flow. High nitrogen concentrations, primarily in the form of NO_3_^−^ due to highly oxic water conditions limiting denitrification, are commonly observed in other carbonate systems (Paytan et al. 2006; Knee et al. 2010; Garcia-Solsona et al. 2010; Hernández-Terrones et al. 2011; Slomp and Van Cappellen 2004; Rodellas et al. 2018; Bejannin et al. 2020; Wang et al. 2018, 2021; Santos et al. 2021). Generally, this elevation is a consequence of anthropogenic nitrogen inputs, such as fertilizer and wastewater. The losses observed in transects B and D might be mitigated by biological activity, the mixing of NO_3_^−^ poor lagoon water (Capone et al. 2008; Rodellas et al. 2018), sedimentation processes, or the absence of groundwater enriched with this ion. However, because nitrate concentrations showed a significant correlation with salinity (*R*^2^= 0.84), this suggests that the physical mixing of SGD with seawater, principally modulated by semidiurnal tides, rather than biological uptake or chemical reactions, is the prevailing process determining nitrogen distribution in coastal water.

In contrast, during the spring tide, all sites were above the mixing line; moreover, some sites showed conservative behavior. This is probably because the sampling was conducted at different tidal times. During the neap tide, we represented both a low and a high tide; in contrast, along the spring tide, sampling began almost at midday, with the tide close to low tide, and ended with the tide beginning the transition toward high tide. The nitrate behavior along the spring tide and the differences between neap tide could be attributed to (1) a greater groundwater contribution during the transition toward high tide and (2) potentially increased tourist activity in the cenote, depending on the time of day. Similar to the neap tide, along the spring tide, nitrate concentrations showed a significant correlation with salinity (*R*^2^= 0.78). This may suggest that physical processes influenced by SGD are the prevailing factors determining nitrogen distribution in coastal waters.

The behavior of ammonium showed enrichment and losses in neap and spring tides. Nonetheless, some stations with conservative behavior were recorded during spring tide (Fig 3A, D), similar to nitrate. Most of the enrichment (neap tide and spring tide) was recorded during the afternoon, however, more enrichment was recorder along spring tide.

Perhaps the tourist activity that takes place in the area contributes to the addition of this ion. No relationship between salinity and NH_4_^+^ concentrations in coastal water samples was observed; however, relatively high concentrations of NH_4_^+^ were indeed measured in high salinity lagoon water (e.g., station G3: 13.9 μM NH_4_^+^ with 37 mg/g of salinity, Station B3: 11.6 μM NH_4_^+^ with 37 mg/g of salinity during neap tide, and station D3: 13 μM NH_4_^+^ with 37 mg/g of salinity during spring tide), corresponding to areas far from SGD influences. In these sites, the enrichment could suggest that there is either production of NH_4_^+^ in the water column, which is unlikely given its aerobic nature (Christensen et al. 2000; Rodellas et al. 2018), or an additional source of these nutrients, most likely inputs from sediments due to diffusion, lagoon water recirculation (Rodellas et al. 2018), and/or resuspension of sediments. The continuous ammonium contribution to coastal waters can be problematic since it can promote macroalgae proliferation (Glibert et al. 2016; Glibert et al. 2018; Wang et al. 2021). Mutchler et al. (2010) recorded the proliferation of *Ceramium* sp. associated with nutrient contribution, originated by anthropogenic activities between 2005 and 2007 in Akumal Bay (2.0 ± 1.9 NH_4_^+^, Mutchler et al. 2007).

The total phosphorus behavior between the neap and spring tide was very contrasting. During the neap tide, only losses were registered. This could indicate a high adsorption rate by sediments because phosphorus is often immobilized through adsorption to mineral surface sites of Fe/Mn oxides (Spiteri et al. 2008) or scavenged by co-precipitation with calcium carbonate (Cable et al. 2002; Santos et al. 2021), and/or active absorption by phytoplankton. In contrast, along the spring tide, losses and enrichment were registered, with most of the enrichment recorded in the afternoon, similar to the ammonium behavior.

Additionally, no relationship between salinity and total phosphorus concentrations in coastal water samples was observed in both periods (*R*^2^= 0.008 neap tide and *R*^2^= 0.014 spring tide), similar to what was reported for the French Mediterranean coastline (Rodellas et al. 2018). This could suggest that SGD is not a significant source of phosphorus to coastal water or that sorption and precipitation occurring in groundwater often forms insoluble inorganic compounds that are sorbed onto rock and particle surfaces (Carreira et al. 2006; Pavlidou et al. 2014).

Along the spring tide, total phosphorus data are more scattered, potentially implying active biological uptake by phytoplankton communities in coastal water. Similar to NH_4_^+^ data, at some sites with high salinity values, high phosphorus concentrations were registered (Station D3: 4.8 μM TP with 36 mg/g of salinity during neap tide, and station D2: 2 μM TP with 35 mg/g of salinity during spring tide), suggesting inputs from sediments due to diffusion and/or resuspension of sediments. However, detailed investigations should be conducted to determine which mechanisms are actually responsible for phosphorus distribution in coastal water.

#### Trophic status and N:P ratio

In the Nohoch-Teek Reef lagoon, the trophic state of each nutrient and the N:P ratio exhibited apparent spatial differences mimicking SGD distribution (Zhang et al. 2020). As for the trophic state, a similar trophic state is observed between spring and neap tide but with different spatial behavior. Different methodologies have been used worldwide to assess the trophic state of various coastal water bodies (Table 3). To the best of our knowledge, for the Mexican Caribbean, this study is the first record evaluating the trophic state. However, for the Yucatan Peninsula, where coastal waters are also influenced by SGD, the trophic condition from Celestún to Ria Lagartos is from good to moderate, indicating a mesotrophic condition. The worst water quality is found in sites influenced by anthropogenic activities (i.e., Sisal, Progreso, and Telchac), while the best water conditions are located at sites where the terrestrial system is included in protected areas (i.e., Celestún, Dzolam Punta Yalkubul) (Herrera-Silveira and Morales-Ojeda 2009; Morales-Ojeda et al. 2010)

**Table 3:** Trophic conditions of some coastal waters influenced by SGD worldwide

Location	Trophic index	Trophic status	Reference
Sekumbu^a^ and Awur^b^ Bay, Indonesian	TRIX (Vollenweider et al. 1998)	Moderate eutrophication^a^ (4–6); Severe eutrophication^b^ >6	Adyasari et al. (2018)
Celestún^a^ and Chelem^b^, Mérida	Trophic Index (Karydis et al. 1983)	NO_2_ mesotrophic^a^, oligo-mesotrophic^b^ NO_3_^+^ mesotrophic^a^, oligo-mesotrophic^b^ NH_4_^-^ mesotrophic^a^, oligo-mesotrophic^b.^ SRP oligotrophic^a,b^	Tapia-González et al. (2008)
Bohai Bay, northern China	TRIX (Vollenweider et al. 1998)	Good to fair (4 < TRIX < 5; 5 < TRIX <6) decreased from 2001 to 2009 and increased after 2009, 2011 and 2012 was fair	Peng (2015)
Kalogria Bay in SE Ionizan Sea	Eutrophication Index (E.I. by Primpas et al. 2010), and BENTIX (benthic macro invertebrate communities)	E.I. : moderate/bad eutrophication status (E.I between 0.38 and 0.85 moderate; E. I greater than 1.51 bad) BENTIX: High ecological quality, station M02, Good to moderate quality, station M19, and Moderate quality, station M20	Pavlidou et al. (2014)
Yucatan coast	TRIX (Vollenweider et al. 1998)	Zone I good to moderate (2.41–5.79); Zone II good to bad (2.75–6.02); Zone III good to moderate (2.43–5.86); Zone IV good to moderate (2.57–5.05)	Morales-Ojeda et al. (2010)
Jiaozhou Bay, China	Water quality index (WQI by Xiao et al. 2014)	Nearshore water: very poor water quality; Central area: good water quality; Bay mouth: poor water quality; Offshore water: good water quality	Zhang et al. (2020)
Yucatan coast	TRIX (Vollenweider et al. 1998); Canadian index for aquatic life (CCMEWQI)	Progreso-Telchac: eutrophication; Dzilam-Las Rocas: mesi-eutrophic; Celestum-Palamar and Ria Largatos-El Cuyo: oligo-mesotrophic	Herrera-Silveira and Morales-Ojeda (2009)

In the Nohoch-Teek reef lagoon, all stations showed nitrate with a mesotrophic TI during neap tide, except for the farthest station to the northeast (oligotrophic B3, low tide). The highest index values associated with the mesotrophic state were recorded in the stations with the greatest influence of the groundwater plume (Fig. 1). A similar trophic state was recorded during spring tide, where nitrate at stations close to the SGD-Teek showed a mesotrophic TI; however, most stations close to the barrier reef and northeast of the SGD (without groundwater influence), such as A6, B, D G3 and I3, showed an oligotrophic TI. This behavior evidences groundwater as the largest nitrate source in the reef lagoon (Slomp and Van Cappellen 2004; Rodellas et al. 2018; Wang et al. 2018), whose direction and scope were recorded predominantly southwest of the reef lagoon, with a maximum extent of ~240 m offshore (unpublished data). Detecting nitrate in an oligotrophic state in the stations close to the reef barrier coincides with the decrease in nitrate concentrations as chlorinity increases (Fig. 3B, E). The high nitrate load reflected in a mesotrophic state and oxygen presence (DO 6.0 ± 1.9 mg/l unpublished data) leads to a simplified nitrogen cycle, with slight nitrate attenuation and high export to the sea (Knee et al. 2010; Null et al. 2014; Montiel et al. 2018). The mesotrophic condition of nitrate was previously recorded by Tapia-González et al. (2008) in the inland area (influenced by SGD and wastewaters) and the mixing zone of Chelem and throughout the Celestún Lagoon (influenced by SGD). These authors indicated that local SGD, chemical characteristics, and perhaps physical processes are essential in promoting trophic status, where spatially the gradients observed are related to the balance of fresh/marine water inputs, residence time (dominated by tide cycles and morphology), biogeochemical processes, and the physical and chemical characteristics of the groundwater (impacts by wastewater) (Medina-Gómez and Herrera-Silveira 2003; Tapia-González et al. 2008).

In contrast, ammonium showed a greater variation in its trophic state in neap and spring tide, with an oligo-mesotrophic behavior. In the Yucatan Peninsula, the lagoons of Celestún and Chelem, sites impacted by anthropogenic activities and influenced by SGD, have exhibited a mesotrophic behavior for ammonium (Tapia-González et al. 2008). Unlike nitrates, the predominantly mesotrophic state of ammonium was recorded northwest of the SGD-Teek during the high tide of the neap tide (transects D and stations B1, B3, A5, G2, G3, H3). The origin of this ion in this zone of the reef lagoon is probably associated with the resuspension processes of the sediment. During the “nortes” season, low air and water temperatures and high wind speeds promote sediment resuspension (Herrera-Silveira and Comin 1998). The TI showed a contrasting behavior concerning the neap tide in the spring tide. At midday (transition of the tide toward low tide), the stations influenced by the groundwater plume showed a mesotrophic ammonium condition; during low tide, the entire lagoon showed mesotrophic conditions except for stations A1, D2, and G1-3, where oligotrophic ammonium was observed. The influx of this ion through SGD and organic matter remineralization accumulated in the groundwater and seawater interface zone may facilitate its availability.

Conversely, phosphorus behavior contrasted between the neap tide and the spring tide. It showed oligo-mesotrophic conditions during the neap tide, registering the mesotrophic condition mainly in the afternoon. In contrast, phosphate compounds showed an oligotrophic condition during spring tide, similar to the oligotrophic status report Tapia-González et al. (2008) for Celestún and Chelem lagoons. Low-phosphate concentrations have been recorded at the SGD (Garcia-Solsona et al. 2010), probably indicating sorption and precipitation along the flow path. It has been reported that phosphate in groundwater often forms insoluble, inorganic compounds that are sorbed onto rock and particle surfaces (Carreira et al. 2006; Spiteri et al. 2007; Rodellas et al. 2015; Santos et al. 2021).

Submarine groundwater discharge has long provided high nutrients to the coastal environment (Paytan et al. 2006; Moore 2010; Kim et al. 2011; Wang 2015; Wang et al. 2018; Santos et al. 2021). The variation in the behavior and trophic state of nutrients in a Reef lagoon influenced by SGD between neap and spring tide is evidenced in this work. Regarding this particular case and according to Beddows (2004), the outflow of the Manatí Cenote will mainly be modulated by sea-level variations; therefore, the constituents transported by groundwater will be influenced principally by such variations and secondary by the anthropogenic activities. In terms of spatial distribution, the variations observed along the lagoon are linked to the balance between fresh and marine water inputs, as well as the distinctive physical and chemical attributes of the groundwater in a karst system. Additionally, biogeochemical activities such as productivity, the breakdown of organic matter, and processes like nitrification and denitrification occurring in specific areas of the system (mixing zone, near SGD, and near barrier reef) play crucial roles in influencing water quality. These processes are closely tied to the duration water remains in a particular location (Medina-Gómez and Herrera-Silveira 2003).

Nitrate influx through the SGD-Teek is reflected in the stoichiometric balance of N:P. The N:P ratio at the SGD-Teek mouth ranged between 41.1 and 68.7, suggesting that SGD may contribute to P-limitation near the discharge, mainly because phosphorus (P) is rapidly removed from groundwater, while nitrogen, in the form of nitrate, is the most prevalent inorganic nitrogen species under freshwater, oxic conditions. The nitrifying bacteria in the soil favor nitrate as the predominant form of inorganic nitrogen (Knee and Paytan 2011). Nitrate is also the most common groundwater pollutant, with main sources being wastewater and fertilizers. The N:P ratios recorded in this study (Table 2) are similar to the minimum ratios reported for river inputs (40:1–140:1) and atmospheric inputs (60:1–120:1) (Ludwig et al. 2009; Markaki et al. 2010) and higher than those observed in the western basin (24:1) and the eastern basin (38:1) of the Mediterranean Sea (Pujo-Pay et al. 2011; Rodellas et al. 2015). And similar behavior has been recorded for the southeastern coasts of the Ionian Sea, eastern China, and the Mexican Caribbean (Pavlidou et al. 2014; Hernández-Terrones et al. 2011; Null et al. 2014; Wang et al. 2018).

In the Yucatan Coast, the N:P ratio exhibits seasonal changes, dropping to less than 15 during the dry season and rising to 60 during the nortes season. This is mainly because aquifer recharge occurs during the previous season, the rainy season, and the winds produce intense coastal currents and tides, which favor sediment resuspension (Morales-Ojeda et al. 2010). In the Nohoch-Teek reef lagoon, it was observed that the groundwater passage in the reef lagoon showed a higher N:P ratio than that of Redfield, mainly in sites influenced by groundwater both in neap and spring tides (along some sites of the transects A, G, H, and I).

However, the N:P ratio rapidly reduced and ranged between 1.4 and 15.1 in the lagoon area with minor influence from the groundwater plume (southeast of SGD). This reduction can be attributed mainly to the drastic nitrogen reduction through biological uptake or a decrease in groundwater influence (Fig. 1). Additionally, phosphorus adsorption and co-precipitation may occur; however, nutrient data indicate a significant reduction in nitrate concentrations between sites influenced by SGD (24.6 ± 10.1 μM NO_3_^−^, Camacho et al. in preparation) and those without SGD influence (6.1 ± 2 μM NO_3_^−^, Camacho et al., in preparation). Phosphorus concentrations, on the other hand, remain constant across all lagoon sites (1.15 ± 0.14 and 1.10 ± 0.12 μM TP with SGD influence and without SGD influence, respectively; Camacho et al., in preparation).

A high N:P ratio at a coastal lagoon influenced by SGD-Teek is expected due to the low phosphate in karst sediments because of its behavior in groundwater and its rapid removal through sorption, precipitation, and biological uptake (Spiteri et al. 2007; Pavlidou et al. 2014; Santos et al. 2021); perhaps the biological uptake to a lesser extent because it has been reported that in karst regions such as the Yucatan Peninsula, phosphorus shows affinity for calcium carbonate, making it less available in the water column (Vuorio and Lagus 2005). An alteration of the stoichiometric equilibrium is one of the most important mechanisms to explain changes in coastal ecosystems, mainly because long-term increases in N:P ratios have been associated with variations in phytoplankton assemblages (Anderson et al. 2002; Wang et al. 2021). SGD are directly linked to eutrophication and harmful blooms in other Asian and Mexican Caribbean regions (Herrera-Silveira and Morales-Ojeda 2009; Liu et al. 2017). However, the behavior and ratio of N:P are significantly influenced by the processes that occur in the mixing zone between groundwater and seawater, where its transformation and removal will be modulated by the flow and turnover rates, as well as redox characteristics (Slomp and Van Cappellen 2004).

### Origin of nitrogen assimilated by octocorals

In tropical carbonated environments such as the Mexican Caribbean, the ammonium and nitrate that enter coastal waters derived from wastewater are rapidly assimilated by autotrophic organisms, forming records of these contributions (Table 4) (Carruthers et al. 2005; Mutchler et al. 2007, 2010; Baker et al. 2013; Sánchez et al. 2013; Camacho-Cruz et al. 2020). As the final destination for the groundwater contained in the Manatí Cenote, the benthic marine environment of the Nohoch-Teek reef lagoon is simultaneously threatened. Even seagrass (*Thalassia testudinum, Halodule wrightii*) and sea fans (*Gorgonia ventalina, Plexaura spp.*) have shown δ^15^N values similar to nitrates contained in groundwater related to anthropogenic contamination (> 7‰; Mutchler et al. 2007, 2010; Baker et al. 2010).

**Table 4:** δ^15^N Isotopic values in octocorals, filamentous algae, and *Thalassia testudinum* across the Mexican Caribbean and the study site

Date	Site	Species	δ^15^N ‰	Source of N	Study
15 July 2019	Cenote	Filamentous algae	5.5	Waste water	This study
	NT A6	*Gorgonia flabellum*	6.1		
	NT A6	*Plexaura cuanquentali*	6.2		
25 January 2020	NT G3	*G. flabellum*	5.9		
	NTG3	*Eunicea succinea*	6.5		
	NT B3	*G. flabellum*	6.2		
March 2002	Puerto Morelos	*Talassia testudinum*	1.7–1.9	N fixation in surface sediments	Carruthers et al. (2005)
May and June 2005	Laguna Lagartos	*Cladophora sp.*	12	Wasted water	Mutchler et al. (2007)
	Pueblo cenote	*Boodleopsis pusilla*	7.8	Wasted water	Mutchler et al. (2010)
May–June 2007	Manati Cenote o Casa Cenote	*Derbesia* and *Polysiphonia aggreagate*	5.5	Waste water	
	Puerto Morelos	*T. testudinum*	0.2–2.5	Pristine condition	Sánchez et al. (2013)
August 2008	Akumal	*G. ventalina*	7.7	Waste water	Baker et al. (2010)
	Mahahual	*G. ventalina*	1.5–3.6	Pristine condition	
2013–2014	Puerto Morelos	*P. nutants*	4.25 ± 0.03		González-De Zayas et al. (2020)
		*G. flabellum*	7.53 ± 0.08	Waste water from SGD	
		*Pterogorgia anceps*	6.55 ± 0.12		
2009–2017	Mahahual	*T. testudinum*	1.9 ± 0.9	Atmospheric deposition	Sánchez et al. (2020)

In pristine conditions, nitrogen isotope values range from 0.2 to 2.5‰ (Sánchez et al. 2013), while atmospheric deposition values are reported at 1.9 ± 0.9‰ (Sánchez et al. 2020). Enrichment in δ^15^N due to denitrification in sediments can also occur (Mariotti et al. 1988), with values of 6 ± 1‰ (Mutchler et al. 2007). However, it is crucial to consider the thickness of the vadose zone and the length of residence times for groundwater in biogeochemically active areas (Cole et al. 2006). In Manatí Cenote, the groundwater discharge rate is high, measured at −5.50 ± 5.21 cm s^−1^ (Beddows 2004). Additionally, if the δ15N resulted from denitrification, a decline in NO_3_^−^ concentration would be expected. Contrary to this expectation, the NO_3_^−^ in this system shows mesotrophic conditions and conservative behavior. Therefore, in this case, the high δ15N values of filamentous algae and octocoral would have resulted from anthropogenic inputs.

The latter are sensitive nitrogen recorders derived from wastewater in various coastal areas of the Mexican Caribbean; the δ^15^N enrichment has been detected more than 1 km offshore on the reef (Baker et al. 2010), suggesting that wastewater-derived nitrogen affects the Mesoamerican Reef System (Ward-Paige et al. 2005; Baker et al. 2010). Contrary to expectations, δ15N does not decrease with depth, but it does when moving away from the source of wastewater (i.e., Yalkul Lagoon) (Baker et al. 2010). However, other factors such as heterotrophic processing of nitrogen can also result in high δ15N values (Carruthers et al. 2005). Nonetheless, octocorals like *G. ventalina* are relatively autotrophic, minimizing the possibility of increasing δ^15^N due to the trophic chain (Baker et al. 2010). In the Nohoch-Teek Reef Lagoon, δ^15^N recorded in octocorals collected from the barrier reef (~600 m offshore) indicated a wastewater-derived nitrogen enrichment (5.5–6.5‰), the leading source pointing to the SGD-Teek. Since octocorals renew slowly (e.g., *G. ventalina*; Yoshioka and Yoshioka 1991), their δ^15^N represents an integrated measure of assimilated nitrogen sources over 6 to 12 months (Baker et al. 2010). This makes it an indicator of annual changes in environmental nitrogen sources.

## Conclusions

The present study shows the complex variation of dissolved inorganic nutrients exchanged between the underground aquifer and the coastal zone. In this environment, atmospheric, oceanographic, and geological conditions will define the variation of this exchange. In the Manatí Cenote system and the adjacent Nohoch-Teek Reef lagoon, the following were observed: (1) In the Manatí Cenote, the behavior of nitrogen compounds and phosphates mostly show systemic enrichment both in neap and spring tide related to tourism activities inside the Cenote. This was more evident in the afternoon when these enrichments were more marked (i.e., ammonium); (2) the trophic state of nitrate and ammonium behaved differently during neap and spring tide; nitrate was eutrophic at the neap tide and mesotrophic at spring tide. Ammonium showed differences before (oligotrophic) and after midday (mesotrophic); (3) the adsorption and coprecipitation capacity of phosphate compounds and their availability are regulated in the system. Despite showing enrichmex`nt, their trophic state was oligotrophic throughout the work and limiting due to a lower N:P ratio than Redfield’s; and (4) nitrogen of anthropogenic origin was detected with an δ^15^N value of 5.5‰.

In the reef lagoon, (1) the behavior of nitrogen and phosphate compounds exhibited apparent spatial differences that mimicked SGD distribution; a similar trophic state was observed between the spring and neap tide, but with a different spatiotemporal behavior; (2) nitrate was mainly enriched, observing an inverse trend with chlorinity; it showed a conservative behavior during spring tide, unlike neap tide; (3) ammonium and phosphorus showed higher enrichment after midday; (4) the trophic state of nitrate was mesotrophic during neap tide and oligo-mesotrophic during spring tide. Ammonium showed an oligo-mesotrophic condition in both neap and spring tide; however, the oligotrophic condition was recorded in the afternoon during neap tide, while this condition was recorded before midday during spring tide; (5) the N:P ratio showed that phosphorus was a limiting compound; and (6) nitrogen of anthropogenic origin (δ^15^N 5.9–6.5‰) was detected ~ 600 m offshore. The exponential growth of anthropogenic activities in karst areas and the lack of infrastructure for wastewater treatment and public regulation enforcement for wastewater discharges threaten the environment. The entry of excess nutrients into the subterranean aquifer and the coastal zone can cause damage to public health and disturb marine ecosystems derived from eutrophication. It is crucial to have an adequate and standardized evaluation method for the level and scope of contamination to manage eutrophication effectively. Analyzing its behavior is an indispensable task that implies an oceanographic, hydrological, and biogeochemical challenge. Based on the present work, an analysis of the trophic state analysis that includes seasonal variation and oceanographic variables is suggested, with periodic monitoring to obtain a complete panorama.

## Supplementary information

ESM 1: Figures S1-S2 (ZIP 756 kb)
