# Supplementary figures and images for: Variability of the trophic state in a coastal reef system associated with submarine groundwater discharge in the Mexican Caribbean
Source: Environ Sci Pollut Res Int. 2024 Mar 20;32(6):3174–93. doi: 10.1007/s11356-024-32818-9 (PMC11832580; doi:10.1007/s11356-024-32818-9)

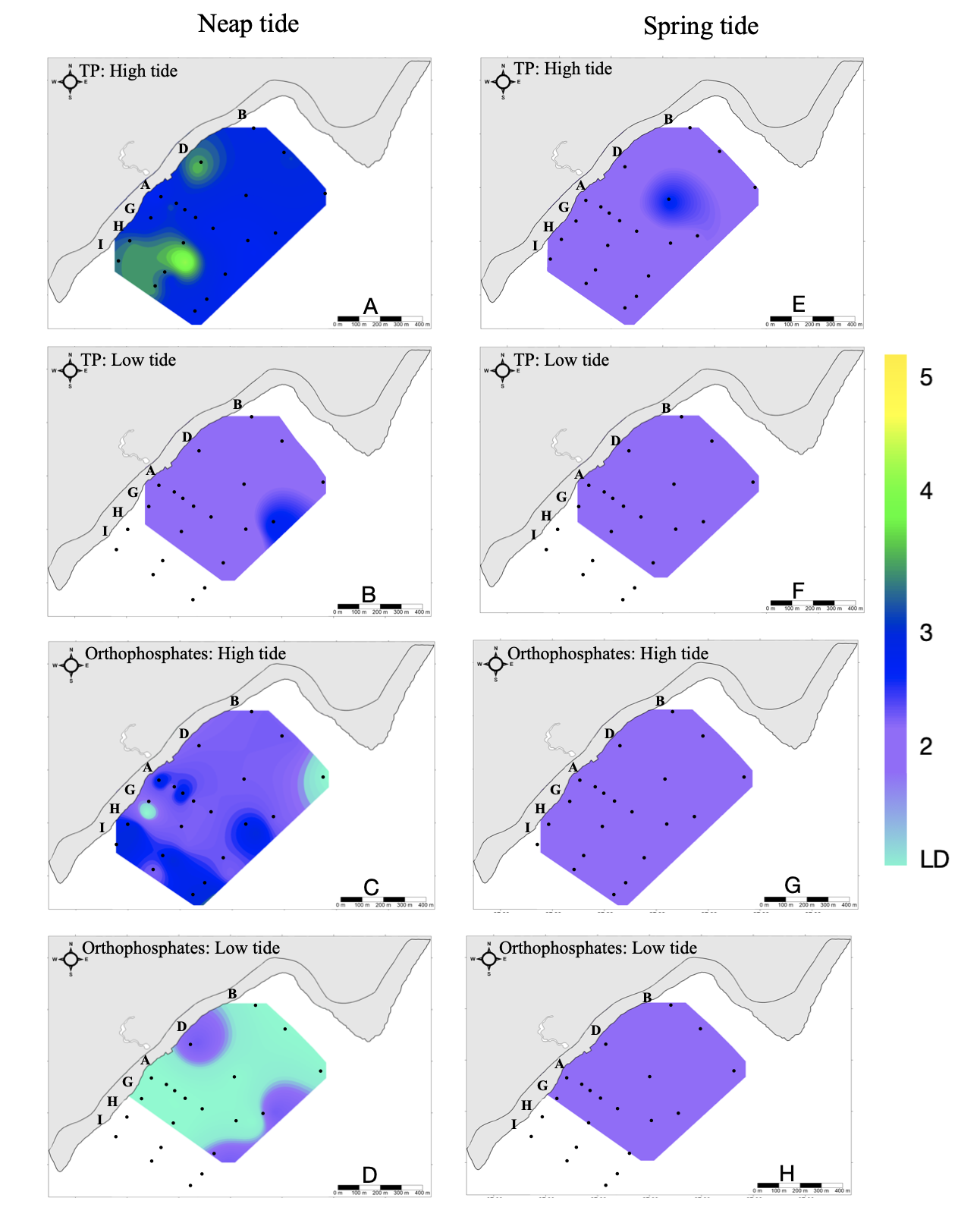

Supplement: Supplementary file 1 — Figures S1-S2 (ZIP 756 kb) [file 11356_2024_32818_MOESM1_ESM.zip › Fig. 1_MS_ESM.png]

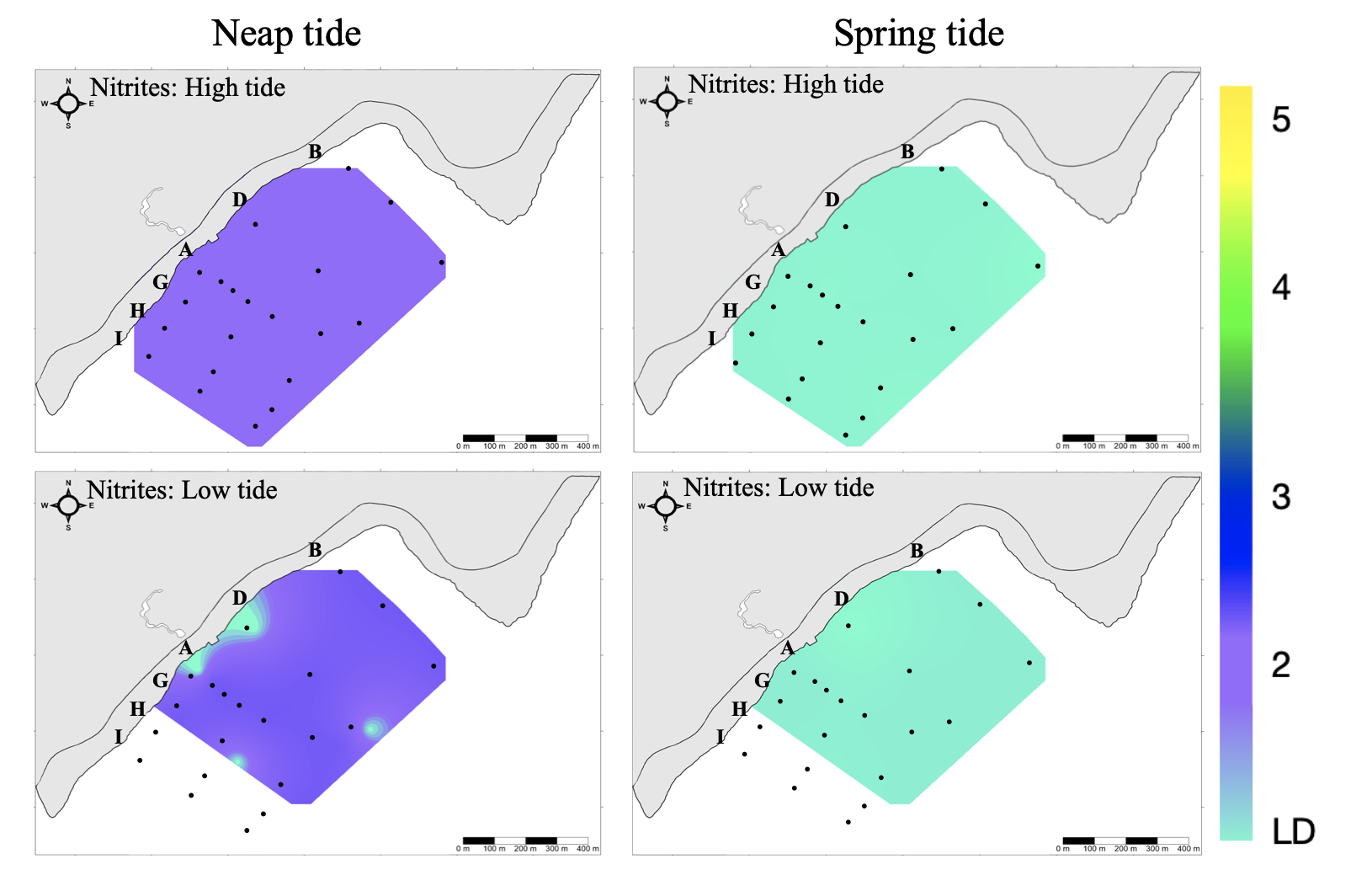

Supplement: Supplementary file 1 — Figures S1-S2 (ZIP 756 kb) [file 11356_2024_32818_MOESM1_ESM.zip › Fig.2_MS_ESM.png]
